# Evaluation of the prototype of a new bracket-positioning gauge

**DOI:** 10.1590/2177-6709.23.2.068-074.oar

**Published:** 2018

**Authors:** Sergio Luiz Mota, Marcio José da Silva Campos, Carina Abrantes Schmitberger, Juliana de Andrade Vitral, Marcelo Reis Fraga, Robert Willer Farinazzo Vitral

**Affiliations:** 1 Universidade Federal de Juiz de Fora, Departamento de Odontologia Social e Infantil (Juiz de Fora/MG, Brazil).; 2 Universidade Federal de Juiz de Fora, Programa de Pós-graduação em Saúde (Juiz de Fora/MG, Brazil).; 3 Universidade Federal de Juiz de Fora, Curso de Graduação em Arquitetura e Urbanismo, (Juiz de Fora/MG, Brazil).

**Keywords:** Orthodontics, Orthodontic brackets, Orthodontics, corrective, Patents.

## Abstract

**Objective:**

The purposes of this study were to present a prototype of a bracket-positioning gauge, which makes vertical inclination of the instrument difficult, allowing a reduction of vertical bracket positioning error, and to test its accuracy in bracket positioning by groups of individuals with different clinical experience and in specific groups of teeth.

**Methods:**

For the testing of the prototype, four groups of six participants each were used: Group 1 was composed of undergraduate students in the dental school, who had no previous experience in bonding orthodontic attachments; Group 2 was composed of orthodontic graduate students in the dental school; Group 3 consisted of orthodontists with a maximum of 5 years of clinical experience; Group 4 comprised orthodontists with more than 5 years of clinical experience. A typodont was simulated with a Class I crowded malocclusion, which reproduced the same occlusal characteristics for all groups to be bonded. All participants were instructed to bond 0.022×0.028-in Edgewise brackets on the labial surfaces of the upper and lower incisors, canines, and premolars at a height of 4 mm from the incisal edge or the labial cusp tip.

**Results:**

Only the mean value of Group 1 showed statistically significant difference in the comparison with the standard measurement. In the groups of teeth, the difference was significant for the premolar and incisor groups.

**Conclusion:**

Clinical experience interfered with the accuracy of vertical positioning of orthodontic attachments. As for the groups of teeth, premolars, followed by canines and incisors had the closest mean values to the standard measurement.

## RESUMO

**Objetivos:**

os objetivos deste estudo foram apresentar um protótipo de posicionador de braquetes ortodônticos que dificulte a inclinação no sentido vertical, possibilitando a redução dos erros de altura no posicionamento desses acessórios; além de testar sua precisão na colagem, realizada por grupos de indivíduos com diferentes tempos de experiência clínica em Ortodontia e em grupos específicos de dentes.

**Métodos:**

para os testes do protótipo desenvolvido, quatro grupos de seis participantes foram formados. O Grupo 1 foi composto por alunos do curso de Odontologia sem qualquer prática com colagem em Ortodontia; o Grupo 2, por estudantes em Ortodontia; o Grupo 3, por ortodontistas com menos de cinco anos de experiência clínica; e o Grupo 4, por ortodontistas com mais de cinco anos de experiência clínica em Ortodontia. Em um *typodont*, foi simulada uma má oclusão Classe I com apinhamento, com as mesmas características para todas as colagens realizadas. Todos os participantes foram instruídos a colar braquetes Edgewise 0,022” x 0,028” na superfície vestibular dos incisivos, caninos e pré-molares superiores e inferiores, na altura de 4mm da borda incisal ou cúspide vestibular.

**Resultados:**

somente a média do Grupo 1 apresentou diferença estatisticamente significativa na comparação com a medida padrão. Nos grupos de dentes, a diferença foi significativa para o grupo dos pré-molares e incisivos.

**Conclusão:**

o tempo de experiência clínica interferiu na precisão do posicionamento vertical do acessório ortodôntico e, quanto aos grupos de dentes, as médias mais próximas à medida padrão foram dos pré-molares, seguidas pelos caninos e incisivos.

## INTRODUCTION

Orthodontic treatment should provide the patient with functional and aesthetic balance between dental, skeletal and facial structures.^1^ ^,^ ^2^ In this process, the correct positioning of orthodontic brackets is of paramount importance for the orthodontic mechanics and treatment results.^3^ ^-^ ^9^ Poorly positioned brackets may result in poor teeth alignment, torque alterations,^10^ ^-^ ^17^ changes in arch length,^18^ distortions in bracket prescription,^19^ as well as occlusal interferences that can compromise masticatory function.^7^ ^,^ ^10^ ^,^ ^18^

For some authors, brackets should be centered on the crown of the tooth,^3^ ^,^ ^20^ whereas others recommend placing the brackets at specific heights for each tooth or group of teeth.^7^ ^,^ ^10^ ^,^ ^13^ ^,^ ^16^ ^,^ ^21^ ^-^ ^25^ Bracket positioning, however, is influenced by the operator and tooth morphology.^2^ ^,^ ^26^ ^-^ ^29^

Studies show that gauges used for bracket placement, when used with the wrong inclination, may interfere with the correct bracket height.^9^ ^,^ ^19^ ^,^ ^28^ ^,^ ^29^ The Boone gauge, one of the most used bracket positioning devices, allows inadequate inclination of the instrument when positioning orthodontic attachments, regardless of the operator’s clinical experience.^30^

The purposes of this study were to present a prototype of a bracket-positioning gauge, which makes undesirable vertical inclination of the instrument more difficult than with other gauges, allowing a reduction of vertical bracket positioning error; and to test its accuracy in bracket positioning by groups of individuals with different clinical experience and in specific groups of teeth.

## MATERIAL AND METHODS

**Figure 1:**
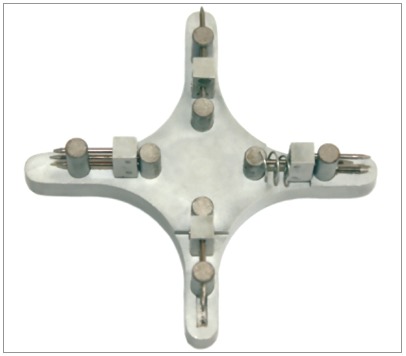
Prototype of bracket-positioning gauge.

**Figure 2:**
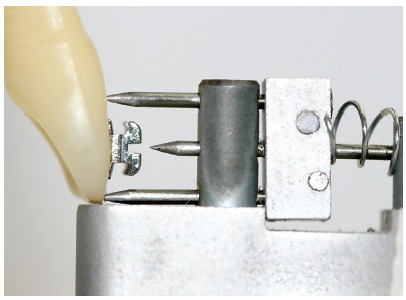
Lateral view of the active point end of the prototype.

**Figure 3:**
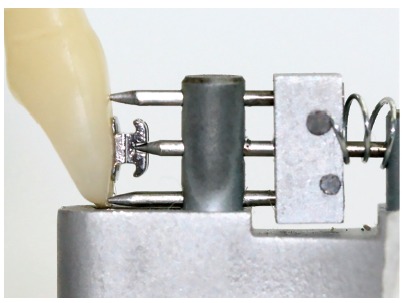
Lateral view of the active point end of the prototype with central pin inserted into bracket slot.

This star-like bracket-positioning gauge was conceived at the Universidade Federal de Juiz de Fora, with funding from FAPEMIG. The gauge was machined and assembled at Robtec industry in the city of Diadema, São Paulo, Brazil. It consists of a star-like metal base with four points, which allow the placement of orthodontic attachments at the heights of 3.5mm, 4.0mm, 4.5mm, and 5.0mm (Fig 1). In order to minimize bracket-positioning error caused by incorrect vertical inclination of the instrument, on each point end of the gauge there are two pins stuck on a sliding base that should contact the labial surface of the tooth above and below the bracket. It has a nickel-titanium coil spring adapted posteriorly to the sliding base, which allows the operator to push the instrument towards the tooth, approaching the central pin toward the bracket (Fig 2). The measuring point end of the gauge should be positioned in a way that the metal base touches the incisal edge or the labial cusp tip of the tooth until the central pin reaches the bracket slot (Fig 3). After the force ceases, the pins and the sliding base return to their original position (Fig 2).

The methodology used in the present study to assess vertical bracket positioning was the same as that described in a previous report by Mota Júnior et al.^30^

In order to test the new gauge, four groups of volunteers were formed according to their clinical experience in orthodontics (Tab 1).

**Table 1:** Distribution of groups of participants, number of participants, and number of teeth in each group.

Group	Participants	Number of participants	Number of teeth
1	Undergraduate students with no clinical experience in orthodontics	6	120
2	Orthodontics graduate students	6	120
3	Orthodontists with a maximum of 5 years of clinical experience	6	120
4	Orthodontists with more tan 5 years of clinical experience	6	120

Comparisons between the groups of teeth were made between incisors, canines, and premolars.

**Figure 4:**
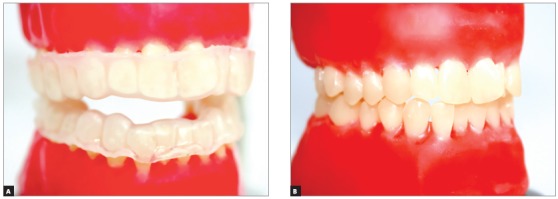
View of typodont with acetate impression tray (A) and with simulated malocclusion (B).

For standardization in assembling a Class I malocclusion for bracket placement (Fig 4A), an acetate impression tray was fabricated to mount the teeth in a typodont (Fig 4B). Teeth were mounted in such a way that the gauge could be used without interference.

Before bracket placement procedure, a video with the instructions on how to use the bracket-positioning gauge was presented. Each participant was instructed to place the 0.022 x 0.028-in Edgewise brackets (380-0021; American Orthodontics, Sheboygan, Wis, USA) on the labial surface of the maxillary and mandibular incisors, canines, and premolars (20 bonded teeth per subject), at a height of 4mm from the incisal edge or labial cusp tip, simulating working clinical conditions. Were also available to the participants: non-surgical procedure gloves, Hollemback carver (CVHL1/29; Hu-Friedy, Chicago, Ill, USA), mouth mirror (M8H; Hu- Friedy), tweezers (DPU17; Hu-Friedy), and bonding tweezers (678-212; Hu-Friedy). For bonding the brackets, Transbond XT composite resin (3M Unitek, Monrovia, Calif, USA) was used and the bracket was cured with a LED curing light for 5 seconds. Neither acid etching of enamel nor a bonding agent was used because teeth were made of plastic and shear bond strength was not under evaluation.

In order to standardize the photographs, teeth were removed from the typodont and inserted into a device with holes filled with self-polymerized acrylic resin (Coldpac Ortho Resin; Yates Motloid, Chicago, Ill, USA) (one hole for each tooth), keeping the mesial surface of the tooth parallel to and facing towards the camera lens. A Canon EOS XSi digital camera of 13 megapixel, with a macro lens of 100mm (2756B001; Canon, Oita, Kyushu, Japan), shutter speed of 1/100 and aperture of f/2.8 was positioned with the camera lens 20cm from the object to be photographed.^30^ A millimeter ruler (CLR6; Hu-Friedy) was inserted in the photographed field to determine the real dimensions of the images in the Keynote software for Mac Os (Version 6.1, Keynote for Mac; Apple, Cupertino, Calif, USA).^30^

**Figure 5:**
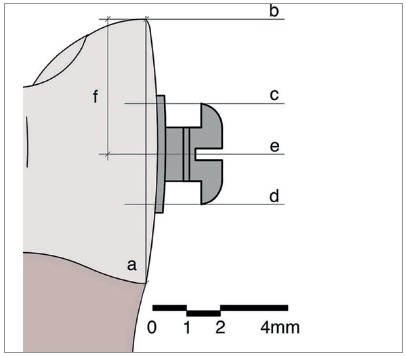
Schematic drawing of the lines used for the evaluation of bracket positioning: line a = representative line of the labial plane (most anterior point of the cementoenamel junction to the most incisal point (or labial cusp tip) of the tooth); line b = perpendicular line to the line a, passing through the most incisal point (or labial cusp tip); line c = perpendicular line to the line a passing through the most superior point of the bracket; line d = perpendicular line to the line a passing through the most inferior point of the bracket; line e = perpendicular line to the line a, equidistant from lines c and d; segment f = bracket placement height.

The determination of the representative plane of the labial surface of the teeth (line a, Fig 5) was done with the mesial view using the union of the most anterior point of the cementoenamel junction and the most incisal point (or labial cusp tip) of the tooth (Fig 5). For the determination of the height of the bonded brackets, the following perpendicular lines to the labial plane were traced: tangent line to the most incisal point (or labial cusp tip) (line b, Fig 5), tangent line to the occlusal border of the bracket wings (line c, Fig 5), tangent line to the cervical border of the bracket wings (line d, Fig 5), equidistant line to lines c and d (Fig 5), representing the geometric center of the bracket slot (line e, Fig 5). In order for any possible rotation or inclination of the bracket not to interfere with the measuring of the vertical positioning of the bracket, the most incisal bracket tie wings and the most cervical bracket tie wings were used to determine the geometric center of the bracket. The bonding distance corresponded to the distance between the points in which lines b and e intercepted the representative plane of the labial face of the tooth (segment f, Fig 5).^30^

### Statistical analysis

To test the calibration of the evaluator regarding the measurements of the digital images, the error of method was calculated using the intraclass correlation coefficient (ICC), in which all measurements were measured twice with a 30-day interval. 

For the statistical analysis the Minitab software for Windows (version 17; Minitab, State College, Pa, USA) was used. The Anderson-Darling test was applied to evaluate the normality of data. For the comparison of the values obtained in each group of participants and for the groups of teeth with the established standard pattern (4mm), the Student’s t-test was performed. Homogeneity of variances was evaluated using Bartlett’s test with a level of significance of 95%.

## RESULTS

The analysis of the calibration procedure showed excellent agreement between evaluations (ICC = 0.995). The sample showed normal distribution (*p*> 0.05) for the Anderson- Darling normality test.

Table 2 presents the values concerning bonding heights in each group of participants (groups 1-4) and the respective p values (Student’s t test) in comparison with the standard measurement (4mm).

**Table 2:** Means, standard deviations, variances, minimum, maximum and p value for each group of participants.

Group	n	Mean	Standard deviation	Variance	Minimum	Maximum	p value
1	120	3.737	0.359	0.129	3.037	4.683	<0.05*
2	120	3.996	0.312	0.097	3.376	4.936	0.900
3	120	3.995	0.303	0.092	2.987	4.743	0.882
4	120	4.039	0.267	0.071	3.026	4.868	0.106

* Statistically significant difference.

**Figure 6:**
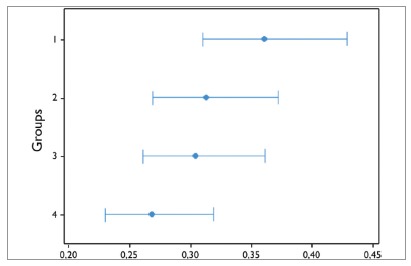
Bartlett’s test to evaluate the homogeneity between the variances in groups 1, 2, 3 and 4.

Figure 6 shows the results of Bartlett’s test, indicating that at least one group of participants showed statistically significant difference when compared to the homogeneity of variance (*p*= 0.014) of the four groups under study.

Table 3 shows the values of the bonding heights of each group of teeth (incisors, canines, and premolars) and the respective p values (Student’s t test) in comparison with the standard measurement (4mm).

**Table 3:** Means, standard deviations, variances, minimum, maximum and p value for each group of teeth.

Group of teeth	n	Mean	Standard deviation	Variance	Minimum	Maximum	p value
Incisors	192	3.877	0.308	0.095	3.026	4.743	<0.05*
Canines	96	4.060	0.312	0.098	3.205	4.743	0.061
Premolars	192	3.948	0.352	0.125	2.987	4.936	<0.05*

* Statistically significant difference.

**Figure 7:**
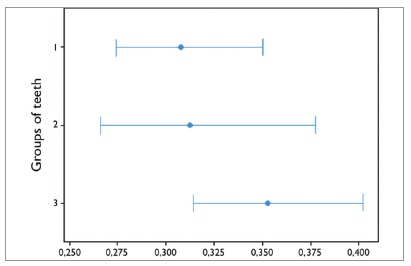
Bartlett’s test to evaluate the homogeneity between the variances in the groups of incisors (1), canines (2), and premolars (3).

Figure 7 demonstrates the results of Bartlett’s test, indicating that there was no statistically significant difference in the comparison of homogeneity of variances in the groups of teeth (*p*= 0.130).

## DISCUSSION

During installation of orthodontic appliance, it is desirable that the brackets be placed in specific positions on the surface of the tooth. Visual acuity^26^ ^,^ ^28^ as well as bracket-positioning gauges^4^ ^-^ ^8^ ^,^ ^27^ ^,^ ^30^ may fail to position orthodontic attachments vertically. The most often used bracket-positionig gauges are the star-like gauge (also known as Boone gauge) and the pole-like gauge (also known as height bracket-positioning gauge).^19^ In case orthodontic attachments are not properly positioned on the surface of the tooth, compensations in the archwire or replacement of orthodontic attachments will be required,^16^ increasing chair and treatment time.^10^ ^,^ ^12^ ^-^ ^14^ ^,^ ^16^ ^-^ ^18^

In order to minimize the errors on the vertical positioning of the brackets caused by the malposition of the gauge, a prototype bracket-positioning gauge, whose main characteristic is to make it difficult for the operator to incline the instrument during its use, was developed. By positioning the upper and lower end points of the star-like stainless steel bracket-positioning gauge on the surface of the tooth, it allows its central end point to be inserted into the bracket’s slot in a perpendicular way to the labial surface of the tooth. The prototype was manufactured with a thicker base than that of the initial project because of the machining process used.

According to Armstrong et al,^28^ one should use the distance from the incisal edge (or labial cusp tip) of the tooth to the center of the bracket as reference for bonding height. By using this reference measurement during the prototype test, only group 1 showed statistically significant difference (*p*< 0.05) in the comparison of the mean (3.737 mm) with the standard measurement (4 mm). Groups 2, 3, and 4 showed means with no significant differences (*p*> 0.05) when compared to the standard measurement: 3.996 mm, 3.995 mm, and 4.039 mm, respectively. When comparing these results with those from tests using similar methodology with the Boone gauge,^30^ it can be noted that, in all groups of participants, bracket placement with the prototype presented results closer to the standard measurement of 4 mm, except the group consisted of undergraduate students with no previous experience in bonding brackets. Besides, this group showed significant greater variance than the other groups (*p*< 0.05) in both studies.

For those subjects with experience in bonding orthodontic attachments (groups 2, 3 and 4), the values of the means did not reveal any relationship with bonding accuracy and clinical experience. Regardless of the clinical experience in Orthodontics, the mean of bonding height was very close to the standard measurement, with no significant differences. Armstrong et al^29^ compared the accuracy of bracket placement by orthodontists and inexperienced dental students and concluded that the vertical accuracy of bracket placement was not related to clinical experience. Mota Júnior et al,^30^ in turn, evaluating four groups of subjects with different clinical experience in bonding brackets, drew the same conclusion.

However, in this type of evaluation the variance in each group seems to be more important than the mean.^30^ The use of this prototype yielded smaller variances in Groups 1, 3, and 4 in comparison with the results from the study with the Boone gauge using similar methodology.^30^ As for clinical experience, the prototype study showed that the longer the clinical experience, the lower the variance in the groups. In the assessment of homogeneity of variances between the groups of subjects, the Bartlett’s test demonstrated that there was statistically significant difference (*p*< 0.05) for Group 1 in relation to the other groups. Despite the fact that no participant in the sample had previous experience with the instrument, clinical experience, associated with the characteristics of the instrument itself, seems to have influenced the better vertical accuracy of bracket placement.

The evaluation of the groups of teeth showed that premolars had the closest means to 4mm, followed by canines and incisors. Despite that, the difference was significant for the groups of premolars and incisors. Although the incisors showed the most distant means to the standard measurement, they showed smaller variance (0.095). For all groups of teeth, the variances found in the present study were smaller than those found by Mota Júnior et al:^30^ 0.115, 0.109, and 0.135, for the incisors, canines, and premolars, respectively.

## CONCLUSION

The prototype demonstrated vertical accuracy for bonding orthodontic brackets.

Clinical experience interfered with vertical accuracy of bracket placement.

As for the groups of teeth, the closest means to the standard measurement were those from the premolars, followed by canines and incisors. There was no difference in the homogeneity of variances.
